# Why gender is relevant to materials science and engineering

**DOI:** 10.1557/s43579-021-00093-1

**Published:** 2021-09-16

**Authors:** Elizabeth Pollitzer

**Affiliations:** Portia, London, UK

**Keywords:** Biological, Bioelectronics, Biomaterial, Biomedical, Environmental impact

## Abstract

For historical reasons science today has substantially more evidence for males and men than for females and women, which means that quality of research and innovation outcomes may often be worse for women than for men. I explore how the gender dimension—a term used to mean effects of biological (sex) and/or socio-cultural (gender) characteristics—fits into new materials research and engineering and especially in nano-materials applications. Horizon Europe expects that grant proposals should include explanation if gender dimension is relevant to the project’s objectives. This paper shows that often the answer should be *yes it is*.

## Introduction

In 2010, a panel of 14 science leaders from across Europe met over a period of three months to examine scientific studies showing if, when, and how (biological) sex and/or (socio-cultural) gender characteristics of the studied population are included in study design, and what such research offers to women and men in terms of evidence and outcomes. The Panel identified widespread historical gender bias and gaps in knowledge and observed specifically that much more data have been accumulated for males and men than for females and women.^[1]^ The cause of this imbalance was the tendency of researchers to assume that ‘science is gender neutral’ (namely, that male–female distinctions among researchers or research subjects are insignificant) and to exclude females as research subject because they were perceived as harder to study for reasons of hormonal influences on their biology and behaviour. Consequently, ‘male’ as the norm came to dominate science knowledge-making, explicitly by excluding females as research subjects, and implicitly by not analysing and not reporting results disaggregated by sex. For instance, at the time of the science leaders’ assessment, 79% of studies in pain research relied on male (rat) model;^[2]^ 75% of cell studies did not distinguish, or report on the sex of the cells studied;^[3]^ and car safety was tested on male crash test dummies, only.^[4]^

The favouring of males as research subject and the implicit use of the ‘male’ as the norm was very likely compounded by the underrepresentation of women in core STEM fields, and especially in the decision-making spheres associated with prioritisation of research, allocation of funding, and in assessments of research excellence.^[5]^ The repercussions of gender bias in science knowledge for women can be easily demonstrated. For instance, even though the risks of cancer from exposure to ionizing radiation are much greater in females than in males (three time higher in girls than in boys), estimations of radiation doses are still based on Reference Man (20–30 years old, Caucasian male, weighing 70 kg and 170 cm tall) and are missing data for children.^[6]^ Likewise, eight out of ten prescription drugs withdrawn from the market in the USA between 1999 and 2001 were more dangerous to women than to men,^[7]^ whilst the analysis of 20 years of car crash accidents data in the USA showed that women drivers had 47% higher risk of serious injury than men had.^[8]^

The realisation that gender equality and research quality are interconnected, and together influence results and outcomes has mobilised new interest in gender issues among policy makers and science leaders, especially in Europe, but also in South Korea, Japan, Canada and USA. In Horizon Europe, for instance, the European Commission expects all proposals to include Gender Equality Plans and to consider if and how gender dimension, a term that covers biological and/or socio-cultural factors, fits into the content and impact of the proposed work, and how it will be addressed if it is relevant.^[9]^ In 2020, the German Research Foundation (DFG) joined this trend by announcing that assessment of the gender dimension will be part of proposal evaluation process.^[10]^ Those new to the term “gender dimension” and its relevance to research quality will benefit from consulting the explanatory materials and scientific references assembled by the EU co-funded Gendered Innovations project accessible through a website hosted at Stanford University.^[11]^

Sex/gender differences start at genetic, molecular and cellular levels^[12]^ and continue their influences through behaviour and socialisation in socio-economic and natural ecosystem. Therefore, understanding when, why and how these differences might influence research results and innovation outcomes becomes directly relevant to materials research and engineering motivated by applications involving humans (e.g., wearables to monitor biomarkers) or intended for deployment in living ecosystems (e.g., in agriculture). Many abstracts listed in the MRS 2021 Spring Meeting Abstract Book reveal interest in biological applications. The summaries do not offer details of the targeted ‘bio’ applications, and often tend to use terms such as “human”, “medical”, or “health”, but the fact that the prefix ‘bio’ appears in 360 pages suggests that it is timely to raise awareness why such studies should consider the relevance of gender dimension, especially as extensive evidence is already available to show that physiology of women is different in many respects from the physiology of men, and that human cells exhibit wildly different concentrations of many metabolites across the sexes. These dimorphisms are demonstrated across all tissues resulting in different molecular metabolic and immune response pathways that are controlled not just by hormones but also by gene expression and environmental exposure.^[13]^

## Materials and methods

### Gender dimension in study design: a materials science perspective

**Figure 1:**
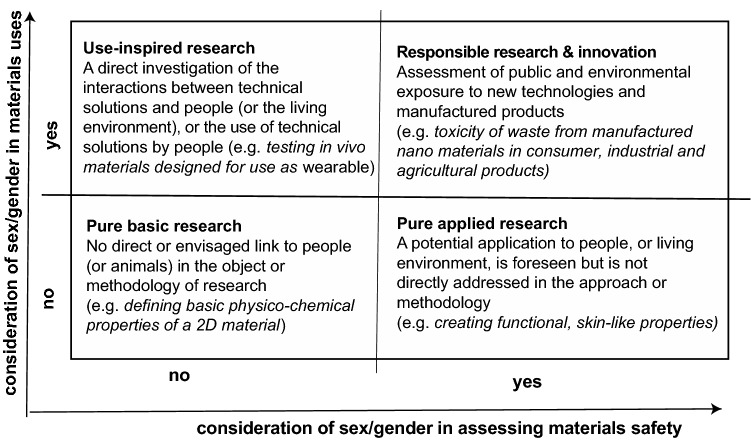
‘Pasteur Quadrant’ for placing gender dimension in materials R&I studies (parts adapted from DFG definitions ^[15]^).

When considering the if’s and the how’s of the gender dimension in materials research it may be helpful to envisage four scenarios: (1) pure basic research with no direct link to people or living beings in the content, e.g., as in studies of physical transformations and chemical reactions of 2D materials with properties useful for electronics products); (2) pure applied research with an indirect application-related link to people/living beings but which is not directly part of the study, e.g., potential use of advanced graphene materials for detecting and removing pesticides; (3) use-inspired research with a direct link to people or living beings whereby analysis of the effects of sex/gender is part of the study, e.g., tissue engineering to examine sex differences and hormone-based effects on tissue homeostasis, repair, and regeneration; and (4) responsible research and innovation (RRI) with a direct impact on eco/socio/economic systems, e.g., toxicological mechanisms and safety hazards of novel nano-materials marketed for use in agriculture/food; paint and coatings; pharmaceuticals; or textiles. The first three of these scenarios have been identified by DFG.^[14]^ The four scenarios are captured in an adaptation of the Pasteur Quadrant framework, shown in Fig. 1.

Only studies firmly located in the pure basic research quadrant can be assumed to be free from the effects of gender dimension. Any spill-overs to application potential and opportunities will, however, benefit from applying a gender lens to make sure that there is no bias in the assumptions, evidence, or interpretation and communication of results.

### Reflections on gender aspects of the studies listed in the MRS 2021 Spring Meeting Abstract Book

The 927 pages of the Abstract Book provide interesting insights into the current trends in materials research and engineering. A search of the text using terms linked to sex/gender, see Table I, shows that terms with the prefix “bio” (e.g., biology, bio-degradable, bio-probes, bio-based, biosensors) appear in 360 pages, which suggests that gender dimension may be relevant to a high proportion of material research. At the same time, the prefix “nano” (e.g., as used in nanotechnology, nano-safety, nanomaterials, nanomedicine) appears in 734 pages suggesting that considerable proportion of nano-application studies with biological connections would benefit from considering the influence of sex/gender differences on results and outcomes.

**Table I:** Results of a search of the abstracts in the MRS 2021 Spring Meeting Abstract Books using sex/gender-related terms.

Number of pages the search term appears	Search term (contextual terms in the abstracts)
734	‘Nano’ (as in nanoparticles, nanoprobe, nanomaterials, nanoelectronics, nanotechnology, nanomedicine, nanoscale)
360	‘Bio’ (as in biology, bio-probes, biodegradable, bioplastic, bio-sourced)
178	Environmental (as in sustainability, assessment, impact, claims, responsibility)
156	Medical (as in bio-, imaging, devices, diagnostics, applications, testing, care)
151	Human (as in being, life, toxicity, health, intervention, understanding)
122	Sustainable (as in practices, future, chemistry, manufacturing, development, design, systems, behaviours, energy, engineering, technologies, actions, materials, electronics, growth, value chains, solutions, production, lab techniques, world, thinking)
114	Toxic (as in toxicology, substances, eco-toxicity, phytotoxic, nature, non-toxic)
102	Health (as in strategies, concerns, care, sector, risks, monitoring, data, outcomes)
89	Wearable (as in devices, electronics, biometric monitoring, clothing, dosimeters)
81	Drug (as in solubilization, delivery, resistance, concentration, screening, addiction)
74	Safety (as in risks, requirements, issues, concerns, bio, applications)
73	‘Skin’ (as in skin-inspired, skin corrosion, electronic skin, human skin, skin cancer, skin surface)
72	Community (as in user, expert, student, effort, wide, -led)
66	Disease (as in diagnosis, cardiovascular, Alzheimer’s, brain, tissue, skin, pulmonary)
61	‘Physiol’ (as in physiological signals, electrophysiological activity, physiological environment, physiological recording, physiological function)
50	Society (as in of professional, and in general)
43	Cancer (as in research, breast, cells, skin, brain, biomarkers, detection, imaging)
36	Patient (as in affected, sepsis, treatment, signal in, population)
36	‘Immun’ (as in immunosorbent, immunofluorescence, immunity, immune system function, immune response, immunogenic)
23	Risk (as in long-term, safety, of failure, of low loss phenomena, nano-particle release, human health)
19	Hazard (as in hazardous chemical use, hazard identification systems, hazardous gases, hazardous substances, hazardous procedures)
14	‘Cardio’ (as in electrocardiography, cardiovascular health, cardiovascular implant, cardiolipin, cardiomyocyte)
13	Physiology (as in electro-, mechano-, patho-, biomedical, detection of)

There are several reasons why focus on safety issues is important. Firstly, the term “hazard” appears in only 19 of the 927 pages of the Abstract Book. Secondly, experts in nanomaterials and nano-products linked to biological effects have identified development of standards for measuring toxicity as a critical need.^[16]^ Thirdly, although some researchers have recognised the need for better safety assessment methods for use in humans and environment, the need to analyse *if* and *how* sex/gender differences might be influencing outcomes has been overlooked.^[17]^ As a rough indication of possible ‘gender blindness’ in assessments of nano-safety is provided by searching Google Scholar with the terms”nanotechnology”, “nano-safety” and “nano-safety gender”, which produce 47,100, 1550, and 75 results, respectively, for each.

## Results

### The special case of nano-biosafety and nanomedicine

It is important that the emerging fields of nano-biosafety and nanomedicine do not repeat the history of gender biases and omissions in science knowledge-making. From the already widely available evidence,^[18]^ it is clear that all the commonly studied biological effects in health-related nano-safety and nanomedicine areas (e.g., reactive oxygen species, skin, immune responses, reproductive and developmental processes, genetic changes, carcinogenesis, and cardiovascular effects) are subject to significant sex-gender difference effects. It is also clear that sex/gender sensitive approaches in research and engineering apply also to non-human organisms, e.g., marine species,^[19]^ and plants.^[20]^

### Exemplifying sex/gender aspects in materials research and innovation

An influential source of advice on conducting gender sensitive research involving human subjects can be found in the SAGER Guidelines devised for preclinical and clinical research which would, therefore, also apply to nano-biosafety and nanomedicine research.^[21]^ For instance, the guidelines recommend that “Authors should report how sex and gender were taken into account in the design of the study, ensure adequate representation of males and females and justify reasons for the exclusion of males or females. Methodological choices about sex and gender in relation to study population and analytical approach should be reported and justified in the same way as other methodological choices.”

Below are a few specific examples of research used to show why adopting methods of sex/gender analysis is relevant to materials research and engineering.

#### X-ray detectors

The Demchyshyn et al.^[28]^ research involves development of ultra-flexible, low-cost, and highly sensitive high energy radiation detectors, which they propose could be of great interest to the fields of medical diagnostics, dosimetry, industrial inspection, security, and because of low weight and high conformability X-ray wearable dosimeters would be appealing to astronauts, nuclear power plants, and laboratory workers, as well as for imagers used in structural inspection and cultural heritage preservation.

Applying gender lens would help reveal a number of efficacy-related issues, such as: (1) important sex/gender differences in the damaging effects of ionizing radiation in female and male tissues; (2) current dosimetry standards are based on inadequate data that are missing evidence for children; (3) death rates among girls following exposure to ionizing radiation is three times higher than in boys; and (4) lack of knowledge about the cumulative effects of repeated low dose exposures to ionizing radiation e.g., from flying at high altitudes, visits to the dentist with X-ray checks; and uses in medical diagnosis.^[22]^ Gender analysis would help also identify opportunities for new markets for such wearables. It would be wonderful for frequent flyers and those experiencing repeated medical uses of X-rays to be able to monitor the total exposure to X-rays over lifetime. Moving in these directions would shift the engineering focus into the use-inspired application quadrant and also to the responsible research and innovation quadrant, thus improving quality of outcomes.

#### Lab-on-skin

Yiran and Wei^[29]^ are developing flexible electronics platform for wearable and flexible sensors for continuous and non-invasive molecular analysis in sweat, tears, saliva, interstitial fluid, blood, wound exudate as well as exhaled breath.

Applying a gender lens would reveal evidence of important sex differences in the basic skin physiologies of women and men, and the well documented sex differences in the diseases mentioned in the abstract (i.e., those linked to metabolic disorders), which are organ- and parameter-specific. For instance, the metabolic profiles of women and men differ in the levels of concentration that many metabolites are produced.^[23]^ This means that the value of “abnormal” must be calibrated separately for women and for men, and that monitoring must be responsive to not just sex differences but also age, since the functioning of the metabolic system changes over lifespan. These considerations would move the investigations into the use-inspired research quadrant and potentially to the RRI quadrant if impact on public health system is also considered, thus increasing the relevance and quality of the outcomes.

#### mHealth biosensors

Wei^[30]^ is developing telemedicine platform for wearable sensors that have the potential to provide rapid, non-invasive, and in-home health monitoring by real-time analysis of biomarkers in human sweat and saliva communicated over Internet. The problem to overcome is that most current biosensors suffer from low sensing accuracy for low-level analyte detection in biofluids and are difficult to fabricate on a large scale. Such wireless platforms could be useful for the rapid COVID-19 test that could provide information on infection status, severity, and immunity.

Applying a gender lens to the development of mHealth biosensor platforms would ensure that women and men are equally included in the tests (use-inspired research quadrant), and in the context of the responsible research and innovation quadrant recognise that because women make up the majority of health workers they should be involved in the innovation process, and, also, that engineering low-cost telemedicine devices could help contribute to the achievement of SDG3 (health and wellbeing) of the UN Sustainable Goals Agenda 2030.

#### Mechano-sensors for physiological signal detection

Kang et al.^[31]^ explore the observation that in nature, spiders sense extremely small variations in mechanical stress using crack-shaped slit organs near their leg joints. Sensors mimicking these mechanisms offer promising application potential in the aspects of sensitivity, stretchability, durability, visualizing, and multi-functionality directed at physiological stimuli such as strain, pressure, and torsion. These researchers envisage that such materials offer advantages to the biomedical applications with complex strain and pressure effects and behaviours as in jaw rehabilitation devices for the neck and head cancer patients.

If such sensors are intended for application on human skins, applying gender lens would help take into account the evidence that men's and women's skins differ in hormone metabolism, hair growth, sweat rate, sebum production, surface pH, fat accumulation, serum leptins, etc.^[24]^. These differences may influence efficacy of the sensors for women and men by not taking into consideration sex differences in skin’s resistance to physical and chemical interactions, potentially related to more frequent reporting of skin sensitivity by women compared to men. Attention to gender dimension would make the investigation part of the use-inspired research quadrant and therefore improve quality of outcomes.

#### Life-like robot behaviour

Mazzolai and Laschi^[32]^ aim to develop life-like robot behaviour by drawing on the lessons learnt from living beings that the physical body has an important role in shaping intelligence. Behaviour is not only controlled by the computation happening in the nervous system but emerges from the interaction of the body with the environment. It then depends on the physical properties of the body itself, on its morphology, on the environment it is operating in.

Applying gender lens would ask if memories stored and mediated through the interactions between the body and the environment are different for women and men and if so, how these differences can be encoded and used for instance in biosensors developed to help stroke victims (more frequent and more severe among women than men) to maintain balance when standing. Another much needed application could be to improve design of crash test dummies to better represent to morphological and biomechanical properties of male and female body and as a result to improve safety of cars for all. Moving in this direction would expand the scope of the study to use-inspired and RRI quadrants.

### Reflection on gender equality in participation

The topic of gender equality is distinct from the topic of gender dimension in that it is about unequal participation of women and men as researchers, decision makers and science leaders. Historically, men have been in a majority at all levels in most STEM fields, with the exception of life sciences, where women have been equally represented up to PhD level. Achieving gender balance in participation is not just a matter of social justice. Research has shown that the benefit of more equal participation is improved collective intelligence,^[25]^ cognitive diversity in problem solving,^[26]^ and more realistic understanding of context,^[27]^ all of which help make research and innovation both relevant and responsible to society. Gender equality is today often connected to diversity (recognition of ethnicity, age, educational status, etc.), and inclusion (recognition of personal identity) issues, which further strengthen opportunities to make science responsible and relevant to societal needs.

There is no scope in this article to explore gender equality issues in materials research and engineering, however, it would be of interest to consider in the future if there are differences in women’s participation across the four Pasteur Quadrants. In addition, given the multidisciplinary aspects of materials research and innovation and the strong interest in bio-related applications, it would be of interest to explore ways to attract to careers in materials science and engineering the talented women graduating in life science in large numbers.

## Discussion

The examples above provide but a snapshot of the current trends in materials research and engineering. Nevertheless, they offer some important insights into how gender dimension can fit into the research itself and drive technological innovation in materials science. The bio-related applications create an important target for advancing methods of sex/gender analysis into materials research and engineering. It is both timely and opportune to raise awareness of potential gender biases and related safety issues linked directly to sex/gender difference effects, especially since scientific evidence supporting such strategy is already available. The timing is important because in Horizon Europe, the European Commission has adopted a policy requiring that proposals include analysis of gender dimension. Similar policy has been adopted in 2020 by the German Research Foundation, and earlier by other funders. Given the high interest in and the rapid growth of nano-biosafety and nanomedicine it would make good sense to also consider if new career pathways could be created enabling women graduating in life and medical sciences, where they are in a majority, to consider transferring their careers into materials research and engineering areas, especially related to health.

As a publisher and curator of knowledge, the Materials Research Society have their own opportunity to prevent gender bias in how studies are reported and communicated by for instance adopting an appropriate editorial policy in their journals, which many journals have already done. Such a policy may be based on the criteria used by DFG. As a publisher MRS can also promote achieving gender balance among editors and inviting studies with a sex/gender perspective in future conferences.

## Data Availability

There is no associated data for this paper.
